# Duplex ultrasound after endo revascularisation (DUSTER): protocol for a randomised controlled feasibility trial

**DOI:** 10.1136/bmjopen-2025-101137

**Published:** 2025-08-10

**Authors:** Nishita Gadi, Carmel Moore, Karen Hayden, Jufen Zhang, Georgina Beetar, Steven Rogers, Clayre Smith-Ball, Alun Davies, Ankur Thapar

**Affiliations:** 1 Mid and South Essex Vascular Unit, Mid and South Essex NHS Foundation Trust, Basildon, Essex, UK; 2 Clinical Trials Unit, Anglia Ruskin University, Chelmsford, Essex, UK; 3 Manchester Academic Health Science Centre, Manchester, UK; 4 Manchester University NHS Foundation Trust, Manchester, UK; 5 The Limbless Association, Basildon, England, UK; 6 Department of Vascular Surgery, Imperial College London, London, UK; 7 School of Medicine and Medical Technology Research Centre, Anglia Ruskin University, Chelmsford Campus, Chelmsford, Essex, UK

**Keywords:** Ultrasound, VASCULAR SURGERY, Follow-Up Studies

## Abstract

**Introduction:**

Endovascular therapy is the main treatment for chronic limb-threatening ischaemia in the UK. Despite a restenosis risk of 50% over 2 years, reintervention rates are low, potentially resulting in preventable amputations. European guidelines recommend ultrasound surveillance to facilitate early treatment of restenosis. This study will investigate the use of duplex ultrasound after endo revascularisation (DUSTER). The aim is to assess the feasibility, acceptability and impact on clinical decision-making of a 1-year integrated ultrasound surveillance programme after lower limb endovascular therapy.

**Methods and analysis:**

DUSTER is a mixed-methods study. Phase I is a three-site, feasibility, open-label, randomised controlled trial. The standard of care, the control arm, is standard clinical surveillance by a vascular specialist at 1, 6 and 12 months. The intervention arm will receive integrated ultrasound (ankle-brachial pressure index, toe pressure and duplex) plus standard clinical surveillance. Primary outcomes are rates of attendance and completion of ultrasound surveillance tests, as well as the percentage of participants undergoing reintervention for restenosis. Secondary outcomes are limb salvage, amputation-free survival, reasons for amputation, complications, serious adverse events and mortality.

Phase II comprises independent semistructured interviews with intervention arm participants. The interviews will explore barriers and facilitators to ultrasound surveillance and the effect of ultrasound surveillance on patients’ lives.

Phase III has two separate focus groups for participants and clinical stakeholders to identify which outcomes matter most in any subsequent large-scale effectiveness trials.

**Ethics and dissemination:**

This research has been approved by a UK (West Midlands, Black Country) Research Ethics Committee (reference 24/WM/0232) and the Health Research Authority (IRAS 349192). Dissemination of results will be by the DUSTER co-investigators in peer-reviewed journals, to the National Institute for Health and Care Research and to a lay audience via the Mid and South Essex NHS Foundations Trust website.

**Trial registration number:**

NCT06702306.

**STRENGTHS AND LIMITATIONS OF THIS STUDY**

- This is a multicentre study assessing uptake across three diverse geographic areas and patient populations, improving the generalisability of findings.
- The use of mixed methods will allow for a comprehensive understanding of feasibility through the collection of both quantitative outcomes and qualitative insights from participants.
- The study has been co-designed with the Limbless Association, a charity for the limb loss community, ensuring patient and public involvement.
- Feasibility and amputation endpoints are objective measures, not subject to bias.
- Due to the nature of the intervention, participant and clinician blinding is not possible.

## Introduction

 Approximately 4000 people in the UK undergo major lower limb amputation each year due to peripheral arterial disease.^1^ The end stage, chronic limb-threatening ischaemia (CLTI), is characterised by nocturnal foot pain, ulceration and gangrene. If untreated, CLTI progresses to amputation, sepsis and death. Hence, CLTI has been the focus of a UK initiative, which aims to reduce amputation rates by expediting limb salvage interventions.^2^ Amputation can have secondary psychosocial impacts, including social isolation, frustration, anxiety and depression.^3^ The average cost of a major amputation, including inpatient care, rehabilitation and prosthetics, is £15 000.^4^ Additionally, amputees may need to move house, require community care, home and vehicle adaptations or Personal Independence Payments and lose employment opportunities.

Endovascular therapy has become the primary modality of limb salvage in CLTI, with >17 000 procedures performed annually in the UK.^1^ These include angioplasty, stenting and advanced interventions, such as intravascular lithotripsy or drug delivery via balloons or stents. These procedures are successful in over 90% of cases, but over 2 years, half of the target vessels restenose or reocclude, putting limbs at risk again.^5^

A recent meta-analysis examined amputation rates following endovascular therapy.^6^ It revealed that 10% of patients lose their leg within the first year of treatment. Restenosis rates were reported at 33% after 1 year and 46% after 2 years. Despite this, reintervention rates were relatively low: 10% at 1 year and 25% at 2 years. This suggests potential for more reintervention to prevent amputations.

Currently, UK vascular specialists detect restenosis through worsening pain, non-healing wounds or loss of previously palpable foot pulses. Out of 51 UK specialists surveyed for duplex ultrasound after endo revascularisation (DUSTER), 83% used clinical follow-up alone. In contrast, the 2019 European Society for Vascular Surgery guidelines recommend three lower limb Doppler scans within the first year post-treatment, combined with ankle-brachial pressure index (ABPI) and toe pressure measurements, in addition to standard clinical follow-up.^7^

In the National Health Service (NHS), it remains unclear whether patients will attend additional scans, whether all scan components can be completed (given factors such as skin ulceration, oedema or toe amputation) or if these scans trigger treatment of restenosis. Ultrasound is safe, accurate and inexpensive. Every NHS vascular unit is equipped with ultrasound facilities, minimising barriers to implementation. Data from Mid and South Essex and Imperial College Healthcare NHS Trusts indicate high attendance rates for vascular ultrasound (95% in 2022–2023), suggesting that ultrasound surveillance should be feasible. If ultrasound prevented one in five amputations,^5^ it could result in NHS cost savings of £5–7 million per year. However, if ultrasound surveillance was introduced but was ineffective, it would increase sonographer workload, unnecessary reinterventions, complications and costs.

The DUSTER study aims to evaluate the feasibility, acceptability and impact of a 1-year integrated ultrasound surveillance programme for patients following endovascular therapy. It will pave the way for an effectiveness study to assess the clinical and cost-effectiveness of this approach, ensuring the surveillance strategy is streamlined and tested for wider adoption.

## Methods and analysis

### Trial design

DUSTER is a mixed-methods study comprising three phases. Eligibility criteria are shown in table 1.

**Table 1:** Eligibility criteria

Inclusion criteria	Exclusion criteria
Adults (18 years or over) who have had successful lower limb endovascular therapy for chronic limb-threatening ischaemia*†‡	Surgical bypass or endarterectomy alone undertaken
Procedures within the last 3 weeks	Patient unfit for or does not want any future revascularisation
Interventions to iliac, femoral, tibial and pedal vessels alone or in combination	Need for major amputation due to gas gangrene, severe Charcot deformity, extensive tissue loss with unstable foot, severe pedal atherosclerosis on angiography or patient request
Able to give informed consent	Patient unwilling or cannot attend further surveillance
Endarterectomy or cutdown is permissible	Interventions for claudication or acute limb ischaemia or trauma
Participation in other vascular trials will be allowed if there is judged to be no risk of affecting validity of each trial by the chief investigators of DUSTER	Patients unable to take any form of antithrombotic therapy
	Prior leg endovascular therapy at the same anatomical location in the study leg (within 1 year)

* Chronic limb-threatening ischaemia refers to ischaemic rest pain, ulceration or gangrene with arterial imaging confirming peripheral arterial disease. Only one leg per patient is eligible for randomisation in DUSTER.

† Successful lower limb endovascular therapy is defined as <30% residual stenosis in the treated segments and patient leaving hospital without a major (above ankle) amputation on study leg.

‡ Endovascular procedures include angioplasty, stenting, atherectomy, lithotripsy, drug eluting balloon or stent or a combination.

DUSTER, duplex ultrasound after endo revascularisation.

Phase I is a multisite, randomised (1:1), controlled, open-label, two-arm feasibility trial. This trial will follow patients up for 13 months. Phase I is due to commence with recruitment in March 2025 and close with analysis in September 2026.

Phase II consists of independent semistructured interviews with intervention arm participants only, regarding their opinions of ultrasound surveillance. The interviews will be held as per participants’ preference on MS Teams or via telephone, after a minimum 6-month follow-up. The interviews will explore the individual’s quality of life, how they manage daily activities, how they look after their affected limbs, how they feel about surveillance and any impact it may have had on their disease/life and what barriers they experienced in completing ultrasound surveillance.

Phase III will include two separate focus groups of participants and clinical stakeholders to identify which of the outcome measures identified in phase I matter most in a follow-on effectiveness trial. This will help define secondary endpoints for a future trial and Health Technology Assessment application if specific progression criteria are met.

**Figure 1:**
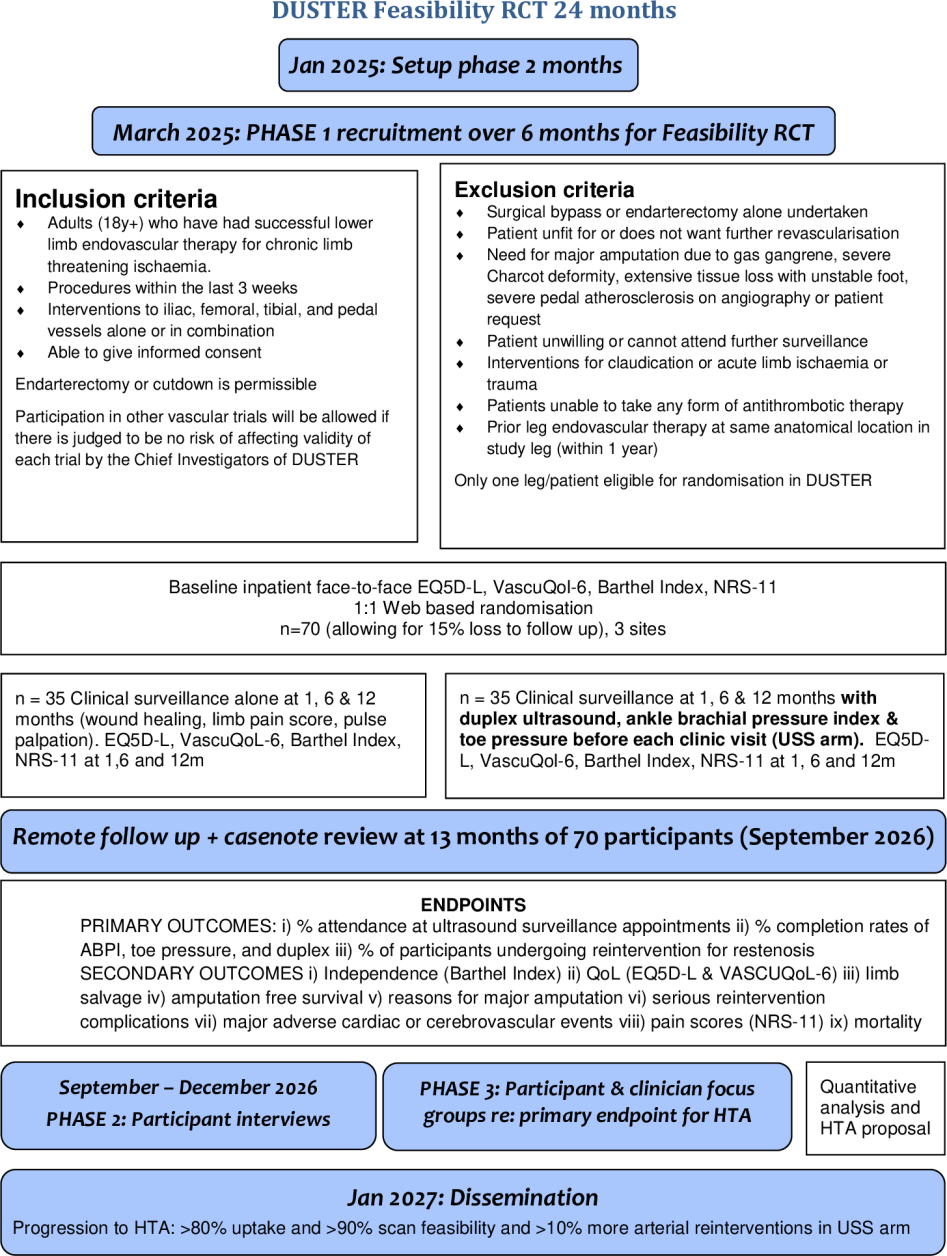
DUSTER, duplex ultrasound after endo revascularisation; EQ-5D-L, European quality of life-five dimensions level; NRS-11, Numeric Rating Scale-11; QoL, quality of life; RCT, randomised controlled trial; VASCUQOL-6, Vascular Quality of Life Questionnaire-6. HTA is an abbreviation for Health Technology Assessment.

Please see figure 1 for a flow diagram of the study design and online supplemental appendix 1 for a Standard Protocol Items: Recommendations for Interventional Trials checklist.

### Primary objective and outcomes

The primary objective is to assess the feasibility of integrated ultrasound surveillance for patients. The following feasibility outcomes at 12 months will be analysed:

1. Percentage attendance at ultrasound surveillance appointments.
2. Percentage completion rates of components of ultrasound surveillance (ABPI, toe pressure and duplex).
3. Percentage of participants undergoing reintervention for restenosis.

### Secondary outcomes

The secondary objective is to provide pilot data for a subsequent trial of clinical effectiveness. For these purposes, secondary outcomes based on core reporting standards will be recorded. These will be measured at 13 months to allow for any reinterventions and their effects triggered from the 12-month visit to be captured. Secondary outcomes are:

1. Limb salvage (freedom from above-ankle amputation).
2. Amputation-free survival.
3. Reasons for major amputation.
4. Serious reintervention complications (unplanned intervention, critical care admission or death).
5. Major adverse cardiac or cerebrovascular events.
6. Mortality and cause of death.

Questionnaire-based secondary outcomes will be self-reported by questionnaire at baseline, 1-month, 6-month and 12-month clinic visits.

1. Quality of life (European Quality of Life-five dimensions level (EQ-5D-L).
2. Disease-specific quality of life (Vascular Quality of Life Questionnaire-6 (VASCUQOL-6)).
3. Independence in activities of daily living (Barthel Index).
4. Pain scores (Numeric Rating Scale-11 (NRS-11)).

### Eligibility

The eligibility criteria are listed in table 1.

### Recruitment

Recruitment will be from three UK vascular units: Mid and South Essex, Imperial College Healthcare and Manchester University NHS Trusts. National Vascular Registry data show an average of six lower limb endovascular procedures per week in total across the three sites. We aim to recruit three participants per week, for a total of 70 over 6 months. Patients will receive a patient information sheet (PIS) during their endovascular procedure hospital stay. Patients will be allowed a further 1-week period to decide to take part. The consent process will be carried out according to the principles of good clinical practice, including a PIS and a signed informed consent form. A model consent form can be found in (online supplemental file 1). Following screening and consenting procedures, patients will undergo a series of baseline assessments or questionnaires to record demographic data and baseline patient-reported outcome measures.

To promote equality, diversity and inclusion, recruitment will be from three diverse sites, two in a multicultural, urban location and another in a semirural, predominantly Caucasian area, situated in the North, South and East of England. Interpreters will be provided for non-English speaking participants, and translation of materials will be provided on request. Travel costs have been incorporated to help overcome problems with transportation. As female patient recruitment has historically been challenging, a link to a female patient and public involvement team member and a female clinical vascular scientist has been made available. It will also be possible to provide participants with members of the research team from different ethnicities.

### Randomisation

Participants will undergo 1:1 web-based randomisation via www.Sealedenvelope.com.

### Study arms

#### Standard care

Standard clinical follow-up at 1 month, 6 months and 12 months with a vascular specialist. This will include wound inspection, pain score (NRS-11) and pedal pulse palpation. A handheld Doppler waveform can be used to supplement the examination if this is standard clinical care at the site. If there is a limb deterioration, further imaging can be requested.

#### Intervention arm

Integrated standard clinical follow-up plus ultrasound surveillance at 1, 6 and 12 months with lower limb arterial Doppler scans performed in hospital, prior to specialist review. Scans will include ABPI, toe pressures and lower limb arterial duplex. It is anticipated that in future trials, one or more of these may be dropped.

Ultrasound results will be reported contemporaneously and those with a treated vessel restenosis of >50% or occlusion, a new inflow or outflow vessel stenosis of >50% or occlusion, an ankle pressure or toe pressure drop of ≥20 mm Hg or absolute pressure values for severe ischaemia (ABPI <0.4 or toe pressure <30 mm Hg)^8^ will be red flagged for clinician attention.

Antithrombotic therapy in both arms will be standardised to first line (aspirin 75 mg once daily and rivaroxaban 2.5 mg two times a day^9^) and second line (clopidogrel 75 mg once daily).^10^ If patients take full anticoagulation for another indication, this should continue, and initiation of additional antiplatelet therapy will be at the operator’s discretion. All patients will require therapy with either high-dose statin therapy (eg, atorvastatin 80 mg) or ezetimibe.^11^ All patients will be offered nicotine replacement therapy.

Reinterventions will be discussed in a local vascular multidisciplinary meeting considering patient wishes, fitness, complexity of procedure and concomitant factors which add to amputation risk (microvascular disease, deformity and infection).

### Sample size

This is a hypothesis-generating feasibility study. A sample size of 70 (35 in each group) will allow us to estimate a 0.85 SD difference in the outcome measures between the groups (90% power, 5% significance and two-tailed). We have accounted for a 15% attrition rate over 12 months from all-cause mortality (12%) and for other reasons (3%). This leaves us with 30 patients per group for analysis of outcomes, which is recommended for a feasibility study with a parallel group design.

### Data monitoring

An independent trial steering committee has been appointed to oversee trial conduct. No data monitoring committee is planned, as this is a feasibility study. A trial management committee will oversee the delivery of the trial. Anglia Ruskin Clinical Trials Unit (ARCTU) will be the coordinating centre. ARCTU will work with the clinical investigators and trial manager on the delivery of the data management in compliance with the applicable regulations.

### Data analysis

As this is a feasibility study, data analysis will be descriptive. A screening log will be used to calculate eligibility rates and participation rates. Withdrawal rates will also be captured. The trial will be reported in accordance with the Consolidated Standards of Reporting Trials guidelines for feasibility trials.^12^

Questionnaire-based outcomes, including EQ-5D-L and VASCUQOL-6,^13^ will be analysed using mixed-effects models for repeated measures with terms for baseline, age, sex, site, intervention group, time (baseline, 1, 6 and 12 months) and an interaction of group and time. Adjusted means and group differences will be presented with 95% CIs. The data will be analysed using an intention-to-treat basis.

For secondary outcomes based on core reporting standards, such as amputation-free survival and limb salvage, the Kaplan-Meier survival curves with 95% CI will be used to present the data by two groups. Event rates will be recorded.

A summary of outcome measures will be described for subgroups of interest, such as gender, ethnicity and recruiting site.

### Data management

Data will be written directly into the case report form (CRF) (source data) and then transcribed into the electronic CRF. Source documents will include original documents related to the trial, to medical treatment and to the history of the participant. Adequate source documentation will be maintained to allow reliable verification and validation of the trial data. CRFs will be in English.

The principal means of data collection from participant visits will be electronic data capture (EDC) in a bespoke database system via the internet. Data will be entered into the EDC system via site personnel. All source data recorded in the CRF will be signed by the investigator or his/her appropriate designee. All changes made following the electronic signing will have an electronic audit trail with a username and date. All trial documentation, including that held at participating sites and the trial coordinating centre, will be archived for a minimum of 10 years following the end of the study.

### Patient and public involvement

Patients and the charity (the Limbless Association) were actively involved in co-designing this study. 11 people with peripheral arterial disease from three hospitals were surveyed in depth about their experience of follow-up, including female and ethnic minority patients. 55% believed ultrasound scans were important in preventing amputation. 91% of patients were happy to attend for 1 year or more of scans, whereas only 36% were happy to attend for 2 years of scans. This has influenced our protocol and criteria for adherence. 100% of patients were willing to undergo reintervention if their scans showed restenosis. A key patient consideration that emerged was independence of activities of daily living, which led to the incorporation of the Barthel Index as a secondary endpoint. The final trial design was reviewed by 14 potential participants from two hospitals: 86% felt comfortable participating in the trial, and 94% felt no ethical, cultural or religious barrier to participation.

### Ethics and dissemination

This research has been approved by a UK (West Midlands, Black Country) Research Ethics Committee (reference 24/WM/0232) and the Health Research Authority (IRAS 349192). Dissemination of results will be by the DUSTER co-investigators in peer-reviewed journals, to the National Institute for Health and Care Research and to a lay audience via the Mid and South Essex NHS Foundations Trust website. Clinicaltrials.gov registration NCT06702306.

## Supplementary material

10.1136/bmjopen-2025-101137 online supplemental file 1
