# Supplementary material for: Duplex ultrasound after endo revascularisation (DUSTER): protocol for a randomised controlled feasibility trial
Source: BMJ Open. 2025 Aug 10;15(8):e101137. doi: 10.1136/bmjopen-2025-101137 (PMC12336602; doi:10.1136/bmjopen-2025-101137)
Supplement: online supplemental file 1 [file bmjopen-15-8-s001.docx]

**Delete this line, then print on Hospital/Trust headed paper**

| Site: MSE / ICH / MUH | Initials: |
| --- | --- |
| Participant Trial ID: | Site Principal Investigator: |

**Duplex Ultrasound Surveillance Trial after Endo Revascularisation - Feasibility RCT**

**IRAS 349192**

**PATIENT CONSENT FORM** Please initial box

1. I confirm that I have read and understood the information sheet (Version 7, 17/04/2025) and have been offered to watch the accompanying video for the DUSTER study and have had the opportunity to ask questions which have been answered to my satisfaction.
2. I understand that my participation is voluntary and that I am free to leave the study at any time without my medical care or legal rights being affected.
3. I understand that relevant sections of my medical records may be accessed by responsible individuals from the research team (at NHS Trusts and Anglia Ruskin University), and relevant regulatory authorities for the purposes of the study and for monitoring and audit purposes. I give permission, provided that confidentiality is maintained, for these bodies to have access to my medical records for this study.
4. I agree to my anonymised data being entered onto a secure database held at Anglia Ruskin University, in accordance with the Data Protection Act 2018, for statistical analysis.
5. I agree to my GP being notified of my participation in this study, including any necessary exchange of information about me between my GP and the research team, e.g. change in medication.
6. If during the study my clinical care team determine that I have lost capacity to provide informed consent, I will be withdrawn from the study and any identifiable data collected to that point would be retained and used in the study.
7. I agree to take part in the DUSTER study.

.

.

**Optional consent section (please initial the appropriate box)**

|  | Give consent | Do not consent |
| --- | --- | --- |
| 8. I agree to being contacted for a follow up interview with researchers from Mid and South Essex NHS Trust to discuss my experiences of ultrasound surveillance *(only for people having extra ultrasound scans).* |  |  |
| 9. I agree to this interview being recorded and stored on NHS computers |  |  |
| 10. I agree to direct quotations from the interview being published as long as they do not identify me. |  |  |
| 11. If I voluntarily withdraw from the study, I am happy for my final cardiovascular outcome data to be collected from the hospital electronic records at the end of the trial. |  |  |
| 12. At the end of the study, I am happy to be contacted by telephone to be invited to an online focus group to discuss what is important to measure from participants points of view in follow on studies. |  |  |
| 13. I give/do not give consent for information collected about me to be used to support other ethically approved research in the future, including outside of the EU. |  |  |
| 14. I give/do not give consent to be contacted in the future with regards to this study, should the study be extended. |  |  |

| Full Name of Participant |  | Date |  | Signature |
| --- | --- | --- | --- | --- |
|  | | | | |
| Name of Person Taking Consent |  | Date |  | Signature |

1. copy for participant; 1 copy for the patient’s medical notes, 1 copy for the site file)
